# Optimization of Time Synchronization and Algorithms with TDOA Based Indoor Positioning Technique for Internet of Things

**DOI:** 10.3390/s20226513

**Published:** 2020-11-14

**Authors:** Kun Zhao, Tiantian Zhao, Zhengqi Zheng, Chao Yu, Difeng Ma, Khaled Rabie, Rupak Kharel

**Affiliations:** 1 Engineering Center of SHMEC for Space Information and GNSS, East China Normal University, Shanghai 200241, China; zqzheng@ee.ecnu.edu.cn (Z.Z.); cyu@cee.ecnu.edu.cn (C.Y.); 51205904080@stu.ecnu.edu.cn (D.M.); 2 Shanghai Key Laboratory of Multidimensional Information Processing, East China Normal University, Shanghai 200241, China; 3 Alibaba Xixi Park, Yu Hang District, Hangzhou 311121, China; bernice.ztt@alibaba-inc.com; 4 Department of Engineering, Manchester Metropolitan University, Manchester M15 6BH, UK; K.Rabie@mmu.ac.uk; 5 Department of Computing and Mathematics, Manchester Metropolitan University, Manchester M15 6BH, UK; R.Kharel@mmu.ac.uk

**Keywords:** indoor positioning, time synchronization, UWB, TDOA, WLS

## Abstract

To provide high-precision positioning for Internet of Things (IoT) scenarios, we optimize the indoor positioning technique based on Ultra-Wideband (UWB) Time Difference of Arrival (TDOA) equipment. This paper analyzes sources of positioning error and improves the time synchronization algorithm based on the synchronization packet. Then we use the labels of the known position to further optimize the time synchronization performance, and hence improve TDOA measurements. After time synchronization optimization, a Weighted Least Square (WLS) and Taylor coordination algorithm is derived. Experiments show that our optimization reduces the average positioning error from 54.8 cm to 12.6 cm.

## 1. Introduction

The number of connected devices is increasing rapidly and will soon continue to grow exponentially. Internet of things (IoT) is a hot research topic, including various application scenarios such as smart transportation, smart security, indoor positioning, etc. Therefore, research on indoor positioning and Location-Based Service (LBS) are receiving increasing attention [1,2]. Thanks to the support of edge computing [3], we can achieve an optimized LBS delivery through the reduced end-to-end latency and load on the transport network.

GNSS is a widely used outdoor positioning technology based on traveling time measurement of the arriving signals. It demonstrates high accuracy, good stability and global coverage. One of the key technologies of GNSS is the time synchronization of the signal transmitter and receiver. When the observation error of the arrival time of the electric signal is less than 3 nanoseconds, the positioning accuracy is better than 1 m. However, we cannot directly transplant GNSS technology to indoor positioning [4] because of the complicated indoor environment, signal interference and equipment cost, etc. In recent years, scholars have proposed different technologies for solving indoor positioning problems [5]. In this article, we focus on the ultra-wideband (UWB), which is widely used in Internet of Things scenarios. UWB signal has low power consumption and strong penetration ability, so it can measure and locate things in complex environments. The bandwidth of UWB is generally several GHz, leading to good time resolution. Therefore, the positioning accuracy of UWB can theoretically achieve the centimeter level.

Among UWB positioning techniques, time-based positioning solutions are dominant, including Time of Arrival (TOA) and Time Difference of Arrival (TDOA) methods. In time-based indoor positioning solutions, time synchronization technology is the cornerstone of high-precision positioning [6]. In TOA-based UWB positioning systems, the clock between the label and the base station needs to be synchronized, and the clock between each base station also needs to achieve synchronization. Meanwhile, UWB positioning systems based on TDOA only require clock synchronization to be maintained among the base stations. In UWB positioning technology based on TDOA, there are three schemes for achieving time synchronization:

1. Synchronization based on a known reference label. Here, the time difference between the signals from the reference label or mobile label and the two fixed base stations is called the single-difference. The difference between the known reference label and another mobile label is defined as the double-difference, which eliminates the clock differences of both the base stations and the labels. This algorithm is inherited from the GPS double-difference observation algorithm and is very common in pseudo-satellite and Locata indoor positioning [7,8]. Both GPS and Locata have base station (or satellite) transmitting signals, but this time synchronization technology is also suitable for label transmitting signals. It is also used in UWB positioning [9]. However, in a complex and ever-changing indoor environment, it is difficult to observe a reference label with a known position without obstruction in the whole process. In addition, base stations and labels need to have the functions of receiving signals and sending signals, and the transmitter must send signals at specified time intervals, which increases the complexity of the device. Therefore, it is only suitable for large and high-cost projects, and is difficult to expand to popular applications.
2. Synchronization based on two-way ranging. In this scheme, both base stations and labels are transceivers. The label (or base station) receives the signal sent by the base station (or label) and forwards it back. After receiving the response signal, the base station (or label) can calculate the distance to the label (or base station) by calculating the time interval between the sent and the rebound signal. Scholars have proposed a scheme to achieve wireless (UWB) clock synchronization by using two-way message exchange [10,11]. The principle of this scheme is simple, and the accuracy is high. However, the base stations and labels must be transceivers. Therefore, the hardware structure of the system is complex, the cost of equipment is high, and the amount of communication is large, and it is difficult to extend it for popular applications, too [12].
3. Synchronization based on timestamp [13]. In this method, the master base station uses the physical layer broadcast function to periodically send messages to the slave base stations. The slave base station uses the arrival time of the message as the reference point of its own clock. The broadcast message contains accurate timestamp. Although this method has less anti-interference compared to other methods, the device of this scheme is simple and the communication volume is small, leading it to be widely used in UWB clock synchronization [14].

## 2. Time Synchronization and Algorithm

In summary, this paper tries to improve the time synchronization scheme with timestamp. The improved scheme is called clock synchronization based on synchronization packets. First, a master base station is selected from among the base stations participating in the positioning, and the other base stations are considered slave base stations [15,16]. Then the master base station periodically sends synchronization packets (including the exact synchronization packet transmission time) to the slave base stations to achieve clock synchronization between the base stations.

The positioning equations based on TDOA measurements have nonlinear characteristics. Therefore, we usually need to convert it into a linear system of equations before solving. Friedlander et al. [17], Schsu et al. [18] and Smith et al. [19] all proposed positioning solution algorithms based on Least Square (LS), but the solutions of the above algorithms are suboptimal. The Chan algorithm [20] uses the double least squares algorithm to give a non-iterative closed solution to the positioning equations. When the TDOA measurement error is relatively small, Chan has the best estimation performance. However, with the increase of TDOA measurement error, the performance of Chan algorithm deteriorates rapidly. The Taylor algorithm is an effective method for solving nonlinear equations, and has the characteristics of high accuracy and good robustness [21,22]. When the initial coordinates of the label have a certain level of accuracy, the Taylor algorithm converges quickly. However, if the initial coordinates of the label are inaccurate, the Taylor algorithm may not converge [23].

This article focuses on UWB indoor positioning technology based on time difference of arrival (TDOA). First, the clock synchronization problem is solved, then the residual error is calibrated, and finally the positioning algorithm is optimized.

### 2.1. Clock Synchronization Modeling

TOA measures the propagation time of the signal from the label to the base station. The one-way observation equation can be expressed as:

(1) $${\Delta t}_{j}^{A_{i}}=t^{A_{i}}-t_{j}=\frac{\left\|{\vec{x}}^{A_{i}}-{\vec{x}}_{j}\right\|}{c}+\left(\tau^{A_{i}}-\tau_{j}\right)+\tau_{m}^{A_{i}}+{\Delta \tau }_{j}^{A_{i}}$$

where ${\vec{x}}^{A_{i}}$ is the position vector, the superscript ($A_{i}$) represents the i-th base station, and the subscript (j) represents the j-th label; $\|{\vec{x}}^{A_{i}}-{\vec{x}}_{j}\|/c$ is the geometric error term and c is the speed of light; $\left(\tau^{A_{i}}-\tau_{j}\right)$ is the clock difference; $\tau_{m}^{A_{i}}$ is the multipath error term which from both label and base station. $\Delta \tau_{j}^{A_{i}}$ are the other error items, such as hardware delay, cable delay, transmission medium delay, etc.

The time difference of arrival (TDOA) between two base stations (A1 and A2) to the same label can be expressed as:

(2) $$\Delta t_{j}^{A_{2}}-\Delta t_{j}^{A_{1}}=\frac{\left\|{\vec{x}}^{A_{2}}-{\vec{x}}_{j}\|-\|{\vec{x}}^{A_{1}}-{\vec{x}}_{j}\right\|}{c}+\left(\tau^{A_{2}}-\tau^{A_{1}}\right)+\left(\tau_{m}^{A_{2}}-\tau_{m}^{A_{1}}\right)+\left(\Delta \tau_{j}^{A_{2}}-\Delta \tau_{j}^{A_{1}}\right)$$

Assuming $\Delta \tau^{A_{2},A_{1}}=\left(\tau^{A_{2}}-\tau^{A_{1}}\right)+\left(\tau_{m}^{A_{2}}-\tau_{m}^{A_{1}}\right)+\left(\Delta \tau_{j}^{A_{2}}-\Delta \tau_{j}^{A_{1}}\right)$, it is the total error of the TDOA-based positioning scheme. Because the clock difference of the label has been eliminated in the single difference, $\left(\tau^{A_{2}}-\tau^{A_{1}}\right)$ is the clock difference between two base stations. When we synchronize the base station clocks, the $\left(\tau^{A_{2}}-\tau^{A_{1}}\right)$ represents the synchronization error, it is usually small and is close to a fixed value. $\left(\tau_{m}^{A_{2}}-\tau_{m}^{A_{1}}\right)$ is the multipath error difference primarily between the two base stations. Multipath errors include extra distance caused by environmental reflections and extra delay in hardware [24]. It is usually at the meter level or more. The base stations are installed at the top of a room with little environmental change, so the multipath error remains relatively unchanged. Therefore, the multipath error difference between two base stations is considered to be a fixed value, which is only affected by the base station. $\left(\Delta \tau_{j}^{A_{2}}-\Delta \tau_{j}^{A_{1}}\right)$ is the difference of other error terms. Hardware delay and cable delay are only relevant to the base station. Due to the short indoor propagation distance, the medium delay is small and can be ignored. Therefore, $\left(\Delta \tau_{j}^{A_{2}}-\Delta \tau_{j}^{A_{1}}\right)$ is considered to be a fixed value.

In summary, $\Delta \tau^{A_{2},A_{1}}$ is the total error difference between two base stations (A2 and A1), which is approximately to a constant. The error calibration method will provide a practical approach to calculate $\Delta \tau^{A_{2},A_{1}}$. If the clock deviation of the two base stations is 1 ns [25], then the TDOA measurement value will also have an error of 1 ns, representing a distance error of 30 cm. Therefore, clock synchronization becomes key to ensuring cm level positioning accuracy.

In general, the base station uses a hardware oscillator to generate the time label. Let the *C(t)* denote the base station clock time. For a perfect clock, $dC\left(t\right)/dt=1$. However, the hardware oscillators all have frequency deviations due to their physical conditions and other factors. It has been found [26] that although the clock frequency is constantly changing, the following formula can be used to approximate the local clock of the base station i:

(3) $$C_{i}\left(t\right)=a_{i}t+b_{i}$$

where $a_{i}$ is the drift rate of the base station clock, $b_{i}$ is the offset of the base station clock, which represents the difference between local time and real time at the beginning. To compare the local clocks of base station 1 and base station 2, it can be expressed as:

(4) $$C_{1}\left(t\right)=a_{12}C_{12}\left(t\right)+b_{12}$$

where $a_{12}$ and $b_{12}$ are the relative drift rate and offset between the clocks of base station 1 and base station 2, respectively. There are two requirements for clock synchronization: (1) the rates of change of the clocks are the same; (2) the offset between the clocks is equal to zero. In short, clock synchronization adjusts the clocks of all base stations in a unified network.

In this paper, we first select a master base station among the base stations participating in positioning, and the other base stations are called the slave base stations. Then, the master base station periodically sends synchronization packets to the slave base stations to achieve clock synchronization between the base stations. In this algorithm, the TOA value recorded by the base station clock is converted into the corresponding TOA value of the master base station clock. Among them, the TOA value measured by the master base station does not need to be converted. Finally, the converted TOA value of the slave base station minus the TOA value of the master base station can get the TDOA value after clock synchronization. The process of obtaining the label TDOA value is:

(1) The master base station sends a synchronization packet k (including the transmission time of the synchronization packet $t_{k}^{M,S}$) to the slave base station. After receiving the synchronization packet, the slave base station saves $t_{k}^{M,S}$ and the local time $t_{k}^{S_{i},R}$ at that moment.
(2) The label sends a positioning packet m. After receiving the positioning packet, each base station saves the local time ($t_{m}^{S_{i},R,T}$ or $t_{m}^{M,R,T}$) at that moment.
(3) The master base station sends a synchronization packet k+1 (including the transmission time of the synchronization packet $t_{k+1}^{M,S}$) to the slave base station. After receiving the synchronization packet, the slave base station saves $t_{k+1}^{M,S}$ and the local time $t_{k+1}^{S_{i},R}$ at that moment.

**Figure 1:**
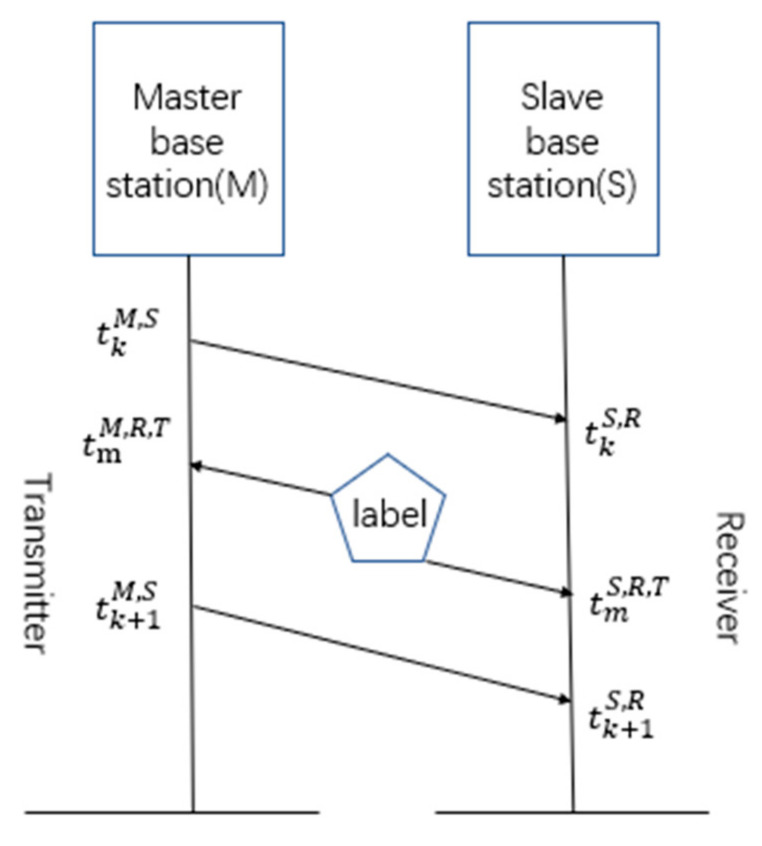
Schematic diagram of the synchronization package.

Both $t_{k}^{S_{i},R}$ and $t_{k+1}^{S_{i},R}$ reduce the propagation time of the synchronization packet between the master base station and the slave base station. The base station coordinates are known and fixed, so the distance between the master and slave base stations is calculated using the point-to-point distance formula. The distance is divided by the speed of light to obtain the propagation time of the synchronization packet between the master and slave base stations. The specific process is shown in Figure 1.

**Figure 2:**
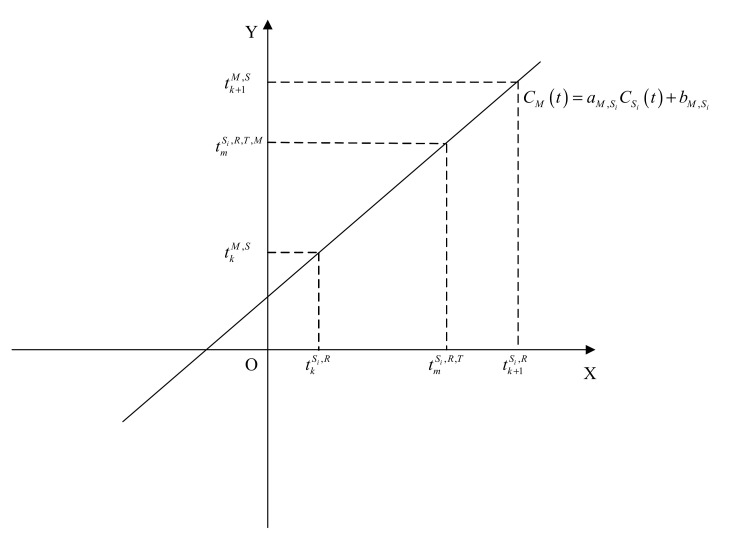
Coordinate graph of the relationship between the master base station clock (*Y* axis) and the slave base station clock (*X* axis).

According to Equation (4), the clock of the master base station M can be expressed by the clock of the slave base station $S_{i}$:

(5) $$C_{M}\left(t\right)=a_{M,S_{i}}C_{S_{i}}\left(t\right)+b_{M,S_{i}}$$

where $a_{M,S_{i}}$ and $b_{M,S_{i}}$ are the relative drift rate and offset between the clocks of the master and slave base stations, respectively. In the positioning system, the base station is equipped with the same clock, and the indoor environment is roughly the same, and the transmission interval of the synchronization packet is also very small, so $a_{M,S_{i}}$ and $b_{M,S_{i}}$ are considered to be fixed values. Therefore, Equation (5) satisfies the nature of a linear function, as shown in Figure 2.

The TOA value recorded by the base station clock is converted into the corresponding TOA value of the master base station clock; the calculation method is as shown in Equation (6):

(6) $$\frac{t_{m}^{S_{i},R,T,M}-t_{k}^{M,S}}{t_{k+1}^{M,S}-t_{k}^{M,S}}=\frac{t_{m}^{S_{i},R,T}-t_{k}^{S_{i},R}}{t_{k+1}^{S_{i},R}-t_{k}^{S_{i},R}}$$

Because both $t_{m}^{S_{i},R,T,M}$ and $t_{m}^{M,R,T}$ are recorded by the master base station, the TDOA value is equal to the TOA value of the base station $t_{m}^{S_{i},R,T,M}$ minus the TOA value of the master base station $t_{m}^{M,R,T}$.

### 2.2. Error Calibration Method

In addition to clock synchronization, there are multipath effects, hardware delays, media delays, and other errors in the positioning process. Therefore, this paper proposes an error processing scheme based on the calibration method, which uses the labels at known locations to determine the error value and stores it in the local server. Then in the following positioning steps, the error value is used to compensate the TDOA value to reduce the positioning error.

**Figure 3:**
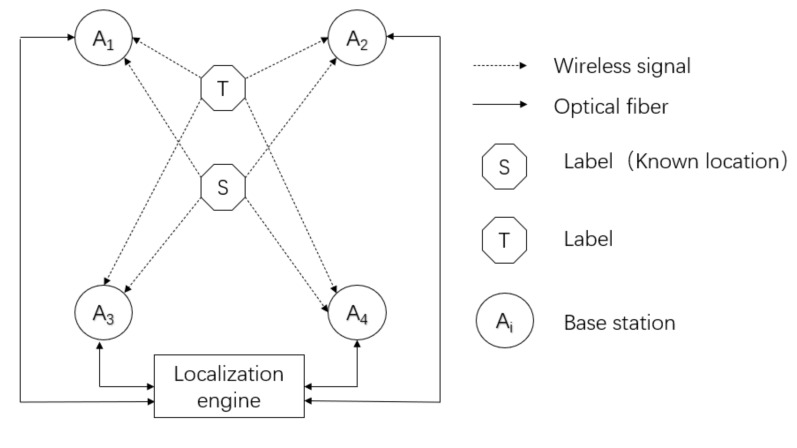
Schematic diagram of error calibration.

In the calibration method, first, a label S (fixed and known coordinates) is required for error measurement. To better explain the error calibration method, a single positioning unit is extracted in the positioning system, as shown in Figure 3. 

The time difference $t_{s}^{A_{i}}$ between the base station $A_{i}$ and the label S is

(7) $$t_{S}^{A_{i}}=\frac{R_{S}^{A_{i}}+l^{A_{i}}}{c}+\tau^{A_{i}}-\tau_{S}$$

where $R_{S}^{A_{i}}$ is the distance between the base station $A_{i}$ and the label S. $l^{A_{i}}$ is the total error of a single positioning (expressed in the form of length). $\tau_{S}$ is the clock difference of the label S clock during the period when it sends the signal. $\tau^{A_{i}}$ is the clock difference of the base station $A_{i}$ clock during the period when it receives the signal. The TDOA value of the same positioning signal measured by base stations $A_{2}$ and $A_{1}$ is expressed in the form of length:

(8) $$\Delta R_{S}^{A_{2},A_{1}}=R_{S}^{A_{2}}-R_{S}^{A_{1}}+c\left(\tau^{A_{2}}-\tau^{A_{1}}\right)+\left(l^{A_{2}}-l^{A_{1}}\right)$$

which is the difference between the base station $A_{2}$ and $A_{1}$ for the same positioning signal of the label, so the label clock difference term is eliminated. $\tau^{A_{2}}-\tau^{A_{1}}$ is the clock difference between base stations $A_{2}$ and $A_{1}$ when they receive the label S signal. The base station has already performed clock synchronization, so $\tau^{A_{2}}-\tau^{A_{1}}$ is eliminated and the remaining is small and classified into $\Delta l^{A_{i_{2}-i_{1}}}$, which can be ignored. Therefore, Equation (8) can be simplified as:

(9) $$\Delta R_{S}^{A_{2},A_{1}}=\left(R_{S}^{A_{2}}-R_{S}^{A_{1}}\right)+\Delta l^{A_{2},A_{1}}$$

where $\Delta l^{A_{2},A_{1}}=l^{A_{2}}-l^{A_{1}}$ represents the difference between the total error (including the clock residual item, multipath error, and other errors) between the base stations $A_{2}$ and $A_{1}$ when calculating the TDOA value of the same label. It is known from Chapter 2.1 that $\Delta \tau^{A_{2},A_{1}}$ is also the difference between the total errors (including the clock residual item, multipath error, and other errors) of the two base stations. Please note that $\Delta l^{A_{2},A_{1}}$ is the length expression of relative total error, and $\Delta \tau^{A_{2},A_{1}}$ is the time expression of relative total error, so that $\Delta l^{A_{2},A_{1}}=\Delta \tau^{A_{2},A_{1}}*c$. The positioning algorithm based on TDOA uses the distance difference to construct a system of equations to achieve the positioning function. Therefore, converting $\Delta \tau^{A_{2},A_{1}}$ to $\Delta l^{A_{2},A_{1}}$ and storing it in the local server will reduce the actual positioning error. From Equation (9), we can get:

(10) $$\Delta l^{A_{2},A_{1}}=\Delta R_{S}^{A_{2},A_{1}}-\left(R_{S}^{A_{2}}-R_{S}^{A_{1}}\right)$$

where $\Delta R_{S}^{A_{2},A_{1}}$ is an observation measurement, which can be obtained by multiplying $\Delta t_{S}^{A_{2},A_{1}}$ and the speed of light. $\Delta t_{S}^{A_{2},A_{1}}$ represents the TDOA value measured by the base stations $A_{2}$ and $A_{1}$. $\left(R_{S}^{A_{2}}-R_{S}^{A_{1}}\right)$ is the difference between the base station $A_{2}$ and $A_{1}$ from the label S. In the error processing, the positions of the set base station and the label are both known and fixed, so $\left(R_{S}^{A_{2}}-R_{S}^{A_{1}}\right)$ can be calculated by the point-to-point distance formula.

In summary, $\Delta l^{A_{2},A_{1}}$ can be calculated using Equation (10). To measure the error calibration value more accurately, this article changes the position of the label many times to form redundant measurements. Then, the best error calibration value is calculated by taking the average of the error calibration values, and storing it in the local server, before finally applying it in actual positioning to reduce the positioning error.

### 2.3. WLS and Taylor Cooperating Algorithm

Based on the error calibration, this paper proposes a Weighted Least Square (WLS) and Taylor collaborative positioning algorithm based on error calibration, which uses WLS to estimate the initial value of the label and uses the Taylor algorithm to determine the label coordinates. The error calibration value is used to compensate the TDOA value of WLS equations and Taylor algorithm.

As shown in Equation (11), the distance between the base station $A_{i}\left(x^{A_{i}},y^{A_{i}}\right)$ and the label $T\left(x,y\right)$ is:

(11) $$R_{T}^{A_{i}}=\sqrt{{\left(x^{A_{i}}-x\right)}^{2}+{\left(y^{A_{i}}-y\right)}^{2}}$$

Equation (10) can be transformed into:

(12) $$\Delta R_{T}^{A_{i},A_{1}}=\left(R_{T}^{A_{i}}-R_{T}^{A_{1}}\right)+\Delta l^{A_{i},A_{1}}\;R_{T}^{A_{i}}=\left(\Delta R_{T}^{A_{i},A_{1}}-\Delta l^{A_{i},A_{1}}\right)+R_{T}^{A_{1}}$$

where $\Delta R_{T}^{A_{i},A_{1}}$ is the distance difference corresponding to the original TDOA value of the base stations $A_{i}$ and $A_{1}$ receiving the label T signal, which can be obtained by multiplying the direct observation (TDOA value) and the speed of light. $\Delta l^{A_{i},A_{1}}$ is the error calibration value of base stations $A_{i}$ and $A_{1}$, as measured in advance using the error calibration method. $\left(R_{T}^{A_{i}}-R_{T}^{A_{1}}\right)$ contains the coordinates of the label T, which is the unknown quantity to be solved.

Set the base station $A_{1}$ as the main base station, and transform Equation (11) to obtain:

(13) $$\begin{array}{c} \left(x^{A_{i}}-x^{A_{1}}\right)x+\left(y^{A_{i}}-y^{A_{1}}\right)y\\ \;=\frac{1}{2}\left({\left(R_{T}^{A_{1}}\right)}^{2}-{\left(R_{T}^{A_{i}}\right)}^{2}\right)+\frac{1}{2}\left({\left(x^{A_{i}}\right)}^{2}+{\left(y^{A_{i}}\right)}^{2}-{\left(x^{A_{1}}\right)}^{2}-{\left(y^{A_{1}}\right)}^{2}\right) \end{array}$$

where i=2, 3, 4 and Equation (13) is a general expression of the geometric distance squared difference in two-dimensional space. Substituting Equation (12a) into Equation (13) gives:

(14) $$\begin{array}{l} (x^{A_{i}}-x^{A_{1}})x+(y^{A_{i}}-y^{A_{1}})y+(\Delta R_{T}^{A_{i},A_{1}}-\Delta l^{A_{i},A_{1}})R_{T}^{A_{1}}=\\ \;-\frac{1}{2}{\left(\Delta R_{T}^{A_{i},A_{1}}-\Delta l^{A_{i},A_{1}}\right)}^{2}+\frac{1}{2}({\left(x^{A_{i}}\right)}^{2}+{\left(y^{A_{i}}\right)}^{2}-{\left(x^{A_{1}}\right)}^{2}-{\left(y^{A_{1}}\right)}^{2}) \end{array}$$

Let $A=\left[\begin{array}{ccc} x^{A_{2}}-x^{A_{1}} & y^{A_{2}}-y^{A_{1}} & \Delta R_{T}^{A_{2},A_{1}}-\Delta l^{A_{2},A_{1}}\\ x^{A_{3}}-x^{A_{1}} & y^{A_{3}}-y^{A_{1}} & \Delta R_{T}^{A_{3},A_{1}}-\Delta l^{A_{3},A_{1}}\\ x^{A_{4}}-x^{A_{1}} & y^{A_{4}}-y^{A_{1}} & \Delta R_{T}^{A_{4},A_{1}}-\Delta l^{A_{4},A_{1}} \end{array}\right]$, $\Delta R=-\frac{1}{2}\left[\begin{array}{c} {\left(\Delta R_{T}^{A_{2},A_{1}}-\Delta l^{A_{2},A_{1}}\right)}^{2}\\ {\left(\Delta R_{T}^{A_{3},A_{1}}-\Delta l^{A_{3},A_{1}}\right)}^{2}\\ {\left(\Delta R_{T}^{A_{4},A_{1}}-\Delta l^{A_{4},A_{1}}\right)}^{2} \end{array}\right]$, $P_{T}=\left[\begin{array}{c} x\\ y\\ R_{T}^{A_{1}} \end{array}\right]$, $\lambda =\frac{1}{2}\left[\begin{array}{c} {\left(x^{A_{2}}\right)}^{2}+{\left(y^{A_{2}}\right)}^{2}-{\left(x^{A_{1}}\right)}^{2}+{\left(y^{A_{1}}\right)}^{2}\\ {\left(x^{A_{3}}\right)}^{2}+{\left(y^{A_{3}}\right)}^{2}-{\left(x^{A_{1}}\right)}^{2}+{\left(y^{A_{1}}\right)}^{2}\\ {\left(x^{A_{4}}\right)}^{2}+{\left(y^{A_{4}}\right)}^{2}-{\left(x^{A_{1}}\right)}^{2}+{\left(y^{A_{1}}\right)}^{2} \end{array}\right]$.

In conclusion, we get

(15) $$AP_{T}=\Delta R+\lambda$$

To reduce the impact of TDOA measurement errors on the positioning results, Equation (15) adds a covariance matrix. After matrix transformation, we get:

(16) $$P_{T}={\left(A^{T}Q^{-1}A\right)}^{-1}A^{T}Q^{-1}\left(\Delta R+\lambda \right)$$

where A and λ are known, and ΔR is the observation matrix after precalibrated propagation delays are corrected. Q is the covariance matrix of observation. Equation (16) can calculate the label coordinates $\left(x_{0},y_{0}\right)$ and $R_{T}^{A_{1}}$.

Equation (16) is a system of equations based on WLS, with small calculation amount and fast speed. When the positioning is in a complex environment such as NLOS, the measurement accuracy of the label TDOA value will be reduced. However, after the error calibration of the TDOA value, the calculation result of Equation (16) can still roughly reflect the true position of the label. Therefore, the solution based on the WLS equation system can meet the requirements of the initial coordinates of the Taylor algorithm. The Taylor algorithm adjusts the label coordinates through the local WLS solution. It has high precision and good stability, but it has a strong dependence on the selection of the label initial coordinates. In summary, this paper proposes a WLS and Taylor collaborative positioning algorithm based on error calibration.

The standard Taylor algorithm is expressed as Equation (17) [27].

(17) h=GΔ+ε

Let, $\Delta =\left[\begin{array}{c} \Delta x\\ \Delta y \end{array}\right]$, $G=\left[\begin{array}{cc} \frac{\left(x^{A_{1}}-x\right)}{R_{T}^{A_{1}}}-\frac{\left(x^{A_{2}}-x\right)}{R_{T}^{A_{2}}} & \frac{\left(y^{A_{1}}-y\right)}{R_{T}^{A_{1}}}-\frac{\left(y^{A_{2}}-y\right)}{R_{T}^{A_{2}}}\\ \frac{\left(x^{A_{1}}-x\right)}{R_{T}^{A_{1}}}-\frac{\left(x^{A_{3}}-x\right)}{R_{T}^{A_{3}}} & \frac{\left(y^{A_{1}}-y\right)}{R_{T}^{A_{1}}}-\frac{\left(y^{A_{3}}-y\right)}{R_{T}^{A_{3}}}\\ \frac{\left(x^{A_{1}}-x\right)}{R_{T}^{A_{1}}}-\frac{\left(x^{A_{4}}-x\right)}{R_{T}^{A_{4}}} & \frac{\left(y^{A_{1}}-y\right)}{R_{T}^{A_{1}}}-\frac{\left(y^{A_{4}}-y\right)}{R_{T}^{A_{4}}} \end{array}\right]$, $h=\left[\begin{array}{c} \Delta R_{T}^{A_{2,}A_{1}}-\left(R_{T}^{A_{2}}-R_{T}^{A_{1}}\right)\\ \Delta R_{T}^{A_{3,}A_{1}}-\left(R_{T}^{A_{3}}-R_{T}^{A_{1}}\right)\\ \Delta R_{T}^{A_{4,}A_{1}}-\left(R_{T}^{A_{4}}-R_{T}^{A_{1}}\right) \end{array}\right]$.

After compensating the TDOA value with error calibration, h in Equation (17) becomes H, $H=\left[\begin{array}{c} \Delta R_{T}^{A_{2,}A_{1}}-\Delta l^{A_{2},A_{1}}-\left(R_{T}^{A_{2}}-R_{T}^{A_{1}}\right)\\ \Delta R_{T}^{A_{3,}A_{1}}-\Delta l^{A_{3},A_{1}}-\left(R_{T}^{A_{3}}-R_{T}^{A_{1}}\right)\\ \Delta R_{T}^{A_{4,}A_{1}}-\Delta l^{A_{4},A_{1}}-\left(R_{T}^{A_{4}}-R_{T}^{A_{1}}\right) \end{array}\right]$. Then, using WLS to process the formula, we can get the improved Taylor algorithm expression:

(18) $$\Delta ={\left(G^{T}Q^{-1}G\right)}^{-1}G^{T}Q^{-1}H$$

where Q is the covariance matrix of observation.

## 3. Experimental Verification

### 3.1. Experimental Environment

**Figure 4:**
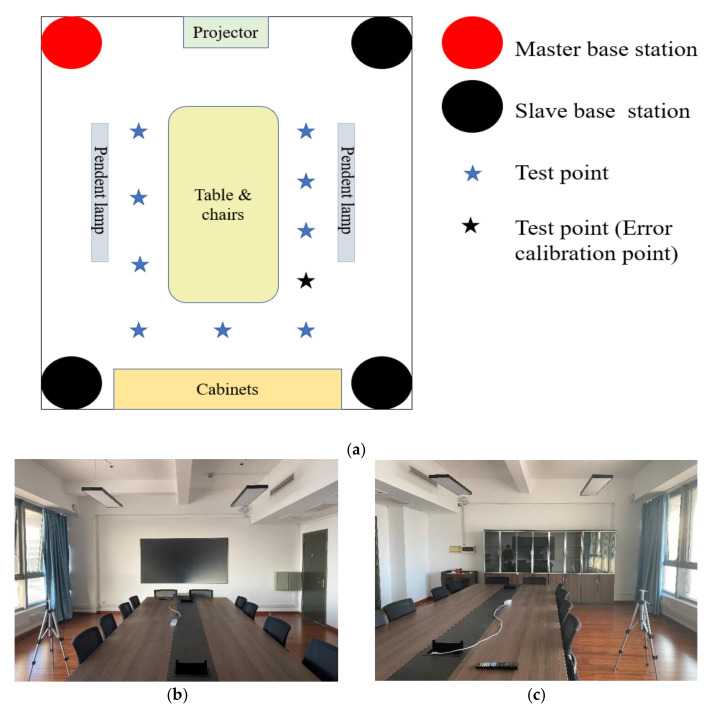
Schematic diagram of experimental environment. (**a**) Diagram of the experimental room; (**b**) Picture of east part of the room; (**c**) Picture of west part of the room.

To illustrate the effectiveness of the error-calibrated WLS and Taylor collaborative algorithm (WLS-Taylor) in real indoor scenarios, experiments were needed. The experimental equipment came from Jiangsu Tangen Technology Co., Ltd. The experimental area was approximately 7 m long and 6 m wide, and was located on the fourth floor of a building of the East China Normal University, being a typical meeting-room environment including one table, chairs, one projector, cabinets, pendent lamps, etc. The experimental environment is shown in Figure 4, with Figure 4a giving the plan of the experimental area, and Figure 4b,c giving the real experimental area.

We used TDOA in the UWB positioning system for the experiment. The system included a hardware and a software component that supported clock synchronization and multiple label access. As shown in Figure 4, the location system had four base stations, of which one was the master base station and three were slave base stations [28].

In the experiment, we preliminarily selected some test points, and sequentially placed the bracket that was used to hold the label at those points. Then, we collected approximately 3000 sets of data without interruption, where the signal of the label was sent at a frequency of 32 Hz. Finally, different algorithms were used to calculate the estimated coordinates of the labels to achieve a controlled experiment. The actual position of the label is measured by an infrared rangefinder, the estimated coordinates were calculated by different algorithms. Afterwards, we calculated the root mean square error between the actual position and the estimated coordinates and the mean of the RMSE (root mean square error) for 3000 data sets which recorded as the positioning error at that point.

### 3.2. Accuracy Evaluation

The average root mean square error between the actual position and the estimated position is used to measure the positioning error of the algorithm.

(19) $$\bar{e}=\frac{1}{N_{p}}\sum_{i=1}^{Np}\sqrt{\frac{1}{N_{t}}\sum_{t=1}^{Nt}e_{i}^{2}\left(t\right)}=\frac{1}{N_{p}}\sum_{i=1}^{Np}\sqrt{\frac{1}{N_{t}}\sum_{t=1}^{Nt}\|P_{i}\left(t\right)-{\hat{P}}_{i}{\left(t\right)\|}^{2}}$$

where $P_{i}\left(t\right)$ represents the actual position of the i-th test point; ${\hat{P}}_{i}\left(t\right)$ represents the estimated position based on the t-th sampling value of the i-th test point; $N_{t}$ and $N_{p}$, respectively, represent the sampling number and the testing number of the test point.

### 3.3. Results Analysis and Discussion

To verify the validity of the WLS-Taylor algorithm, we compared it with the Chan and Taylor (Chan–Taylor) algorithm [29] using TDOA values with and without error calibration.

In the Chan–Taylor algorithm, the output of the Chan algorithm is the initial input of the Taylor algorithm. Then, the Taylor algorithm is sequentially iterted to adjust the coordinates of the label until the sum of the absolute values of the label coordinate change is less than the threshold, outputting the final estimated coordinates of the label.

In the Chan–Taylor algorithm, an extra weighted least squares operation is added to further decrease the perturbation errors of Initial coordinates. In the WLS-Taylor algorithm, we directly used the initial coordinates as the initial value of the Taylor algorithm. The reason for this change is that for the Chan–Taylor algorithm, the primary purpose of the Chan algorithm also meets this requirement, and the calculation amount is smaller than the Chan algorithm because one WLS operation is removed.

The WLS-Taylor algorithm with error calibration uses a pre-calibrated value of error to compensate for the measured TDOA values in the WLS-based equation set and the Taylor algorithm. The results of the WLS-based set of equations were used as the initial values for Taylor and the same calculation of C-T algorithm, allowing us to find the coordinates of the label.

#### 3.3.1. Experimental Analysis of Time Synchronization

**Figure 5:**
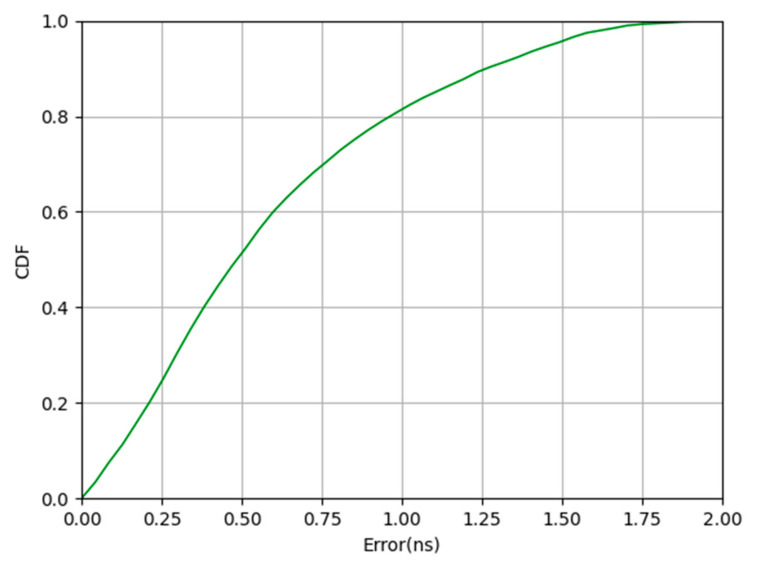
Experimental results of time synchronization with synchronization packets.

We calculated the difference (absolute value) between the 3000 sets of labeled TDOA measurements and the true value to evaluate the performance of the clock synchronization method based on the synchronization package. The TDOA measurement value was obtained by Equation (6). The real TDOA value acquisition is divided into three steps. At first, we calculated the actual distance between the label coordinates and the coordinates of each base station. Second, the distance from the slave base station to the label minus the distance from the master base station to the label was calculated. Third, by dividing by the speed of light, we finally obtained the real TDOA value. Because it is the same system, the base station has the same hardware construction. The clock deviation (absolute value) between one slave base station and the master base station is listed here, and the results are shown in Figure 5.

As shown in Figure 5, the TDOA error is about 1.26 ns when CDF = 90%, and 0.48 ns when CDF = 50%. It can be seen that this synchronization approach is effective.

#### 3.3.2. Experimental Analysis of the WLS-Taylor Algorithm

As shown in Figure 4, the test points are around the table, and we selected one of them as the error calibration point and stored the error scale quantification of the room in local server. The other test points in the room use the error scale quantification for error processing.

**Figure 6:**
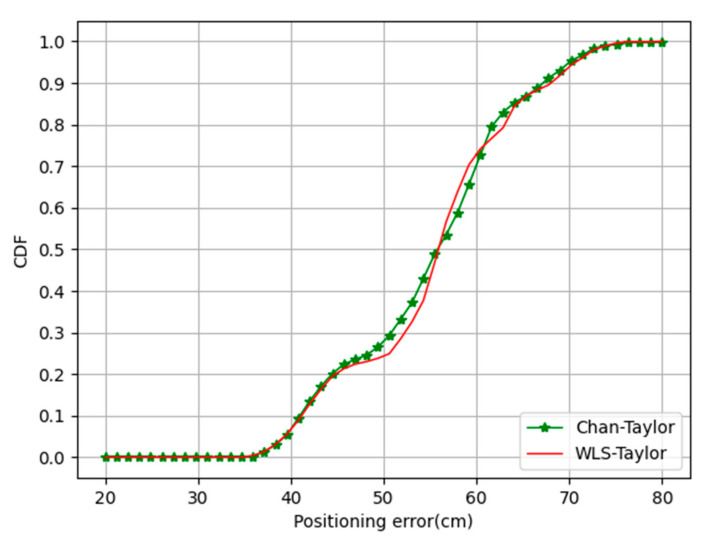
Positioning error of the Chan–Taylor algorithm and the WLS-Taylor algorithm.

In experiments to verify the validity of the WLS-Taylor algorithm, neither the Chan–Taylor algorithm nor the WLS-Taylor algorithm compensated for TDOA values using error scales. The experiment used nine test points to evaluate algorithm performance, except for one error calibration point. The Cumulative Distribution Function (CDF) curves, which are able to completely describe the probability distribution of a real random variable for the positioning results, are shown in Figure 6.

It can be seen from Figure 6 that the positioning accuracy of the WLS-Taylor algorithm is close to that of the Chan–Taylor algorithm. The specific positioning results are shown in Table 1.

As shown in Table 1, the positioning error of the Chan–Taylor algorithm ranged from 35.7 cm to 88.7 cm, with an average positioning error of 54.8 cm and a standard deviation of 9.3 cm, and the WLS-Taylor algorithm ranged from 34.9 cm to 78.8 cm, with an average positioning error of 55.15 cm and a standard deviation of 9.2 cm. This shows that the use of the WLS-Taylor algorithm can reduce the computational complexity while ensuring the accuracy and stability of the final result.

#### 3.3.3. Experimental Analysis of Error Calibration

**Figure 7:**
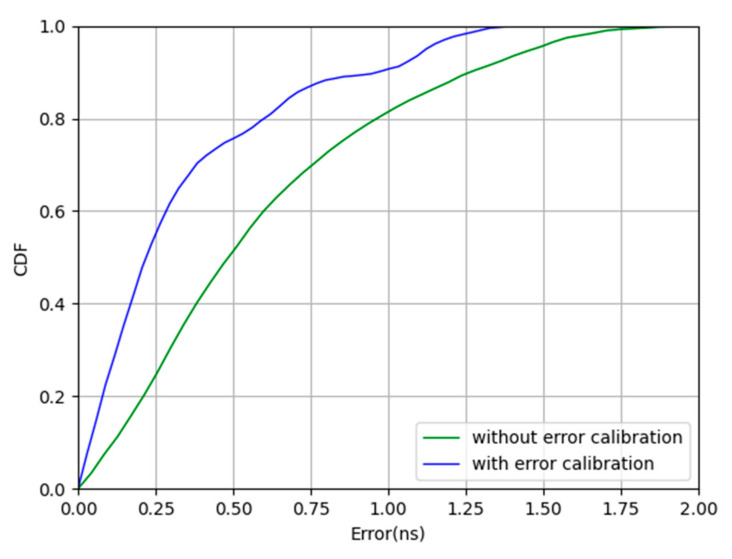
Time synchronization with and without error calibration.

To confirm the effectiveness of the error calibration, the TDOA error with error calibration between one slave base station and the master base station was compared with that without error calibration, as shown in Figure 7.

The experiment was based on clock synchronization, as shown in Figure 7; when CDF = 90%, the blue curve is about 0.97 ns, and the green curve is about 1.26 ns, which is an improvement by 23% when using the error calibration. The CDF curve proves the effectiveness of the error calibration.

**Figure 8:**
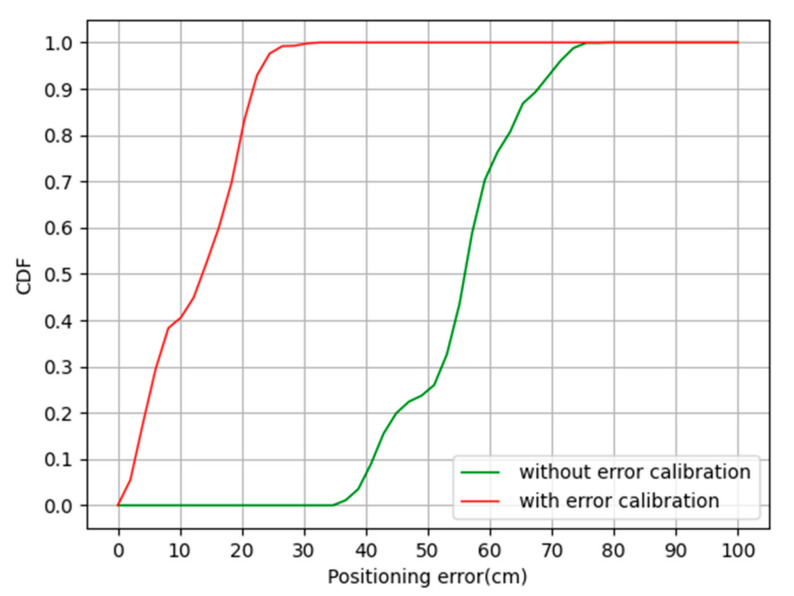
Positioning error improvement by the error calibration.

The green curve in Figure 8 is the result of using the WLS-Taylor algorithm to calculate the estimated coordinates of the label directly without any treatment of the TDOA value; the red curve in Figure 8 is the result of using the error scale to quantitatively compensate the TDOA value and then using the WLS-Taylor algorithm to calculate the estimated coordinates of the label. The error scale quantification and TDOA measurement values are directly added to the algorithm as new TDOA values, and no internal changes are made to the algorithm. Except for 1 error calibration points, the experiment used 9 other test points to evaluate the performance of the algorithm. The CDF curves of the positioning results are shown in Figure 8.

It can be seen from Figure 8 that when CDF = 90%, the positioning accuracy of the W-T algorithm without the use of error calibration is about 67.3 cm; the positioning accuracy of the C-T algorithm with the use of error calibration is about 21.8 cm. It can be seen that the error calibration method improved the positioning accuracy by 67.6%. The specific positioning results are shown in Table 2.

As shown in Table 2, the positioning error of the WLS-Taylor algorithm without error calibration ranged from 34.9 cm to 78.8 cm, with a mean positioning error of 55.2 cm and a standard deviation of 9.2 cm, and the positioning error of the WLS-Taylor algorithm with error calibration ranged from 0.1 cm to 30.7 cm, with a mean positioning error of 12.6 cm and a standard deviation of 7.4 cm. Using the standard deviation to measure the stability of the positioning algorithm, it can be seen that the stability of the WLS-Taylor algorithm improved by 19.6% after using the error calibration method.

## 4. Conclusion

Focusing on the problem that indoor positioning is difficult to achieve high-precision positioning, this paper studied the UWB indoor positioning scheme based on TDOA. The core part was clock synchronization, error calibration and collaborative positioning. With respect to clock deviation between the base stations of UWB positioning system, this paper first proposed a clock synchronization algorithm based on synchronization packets. Then this paper analyzed the principle and error source of TDOA positioning algorithm, and the use of labels with known positions to pre-calibrate errors such as hardware delay and multipath. The error calibration value is stored in the local server to compensate the TDOA measurement value in the actual positioning. Finally, this paper proposed a WLS and Taylor collaborative positioning algorithm based on error calibration. First, the error calibration value is used to compensate the TDOA value, then WLS is used to estimate the initial coordinates of the label and the Taylor algorithm is used to determine the label coordinates.

To obtain a more accurate TDOA value, the clock synchronization method in this paper sends a synchronization packet with a small period. In the next step, algorithms such as Kalman filtering can be introduced to track the clock deviation to reduce the frequency of synchronization packets. This method reduces the calculation load, production cost and volume of the mobile terminal (label), without laying a large number of additional hardware equipment, and without changing the existing hardware equipment, completely relying on software for implementation, strong operability, and easy for popular applications. It has great advantages and business prospects in the indoor positioning application environment where the manager application is the main management for multiple users. In addition, the calibration algorithm has a weakening effect on the multipath effect, but it still has a greater impact on the positioning accuracy and needs further study.

## Figures and Tables

**Table 1:** Experimental results table of the C-T algorithm and the W-T algorithm.

Algorithm	Max (cm)	Min (cm)	Mean (cm)	Standard Deviation (cm)
Chan–Taylor	88.7	35.7	54.8	9.3
WLS-Taylor	78.8	34.9	55.2	9.2

**Table 2:** Experimental result table of error calibration method.

Algorithm	Max (cm)	Min (cm)	Mean (cm)	Standard Deviation (cm)
WLS-Taylor without error calibration	78.8	34.9	55.2	9.2
WLS-Taylor with error calibration	30.7	0.1	12.6	7.4

## References

[1] Zafari F., Gkelias A., Leung K.K. (2019). A Survey of Indoor Localization Systems and Technologies. IEEE Commun. Surv. Tutor..

[2] Del Peral-Rosado J.A., Seco-Granados G., Raulefs R., Leitinger E. (2018). Whitepaper on New Localization Methods for 5G Wireless Systems and the Internet-of-Things.

[3] ETSI Home Page. https://www.etsi.org/deliver/etsi_ts/123500_123599/123502/15.02.00_60/ts_123502v150200p.pdf.

[4] Seco F., Jimenez A.R., Prieto C., Roa J., Koutsou K. A survey of mathematical methods for indoor localization. Proceedings of the 2009 IEEE International Symposium on Intelligent Signal Processing.

[5] Röbesaat J., Zhang P., Abdelaal M., Theel O. (2017). An Improved BLE Indoor Localization with Kalman-Based Fusion: An Experimental Study. Sensors.

[6] Yang Y., Yang K. Time Synchronization for Wierless Sensor Networks using the Principle of Radar Systems and UWB Signals. Proceedings of the 2006 IEEE International Conference on Information Acquisition.

[7] Dai L., Rizos C., Wang J. (2001). The Role of Pseudo-Satellite Signals in Precise GPS-Based Positioning. J. Geospat. Eng..

[8] Li X., Zhao M., Liu Y., Li L., Ding Z., Nallanathan A. (2020). Secrecy Analysis of Ambient Backscatter NOMA Systems under I/Q Imbalance. IEEE Trans. Veh. Technol..

[9] Liu D., Sheng B., Hou F., Rao W., Liu H. (2014). From Wireless Positioning to Mobile Positioning: An Overview of Recent Advances. IEEE Syst. J..

[10] Hach R., Rommel A. Wireless synchronization in time difference of arrival based real time locating systems. Proceedings of the 2012 9th Workshop on Positioning, Navigation and Communication.

[11] Li X., Mengyan H., Liu Y., Menon V.G., Paul A., Ding Z. (2020). I/Q Imbalance Aware Nonlinear Wireless-Powered Relaying of B5G Networks: Security and Reliability Analysis. IEEE Trans. Netw. Sci. Eng..

[12] Qin S., Sihao Z., Xiaowei C. (2018). An Indoor Localization System Using Wirelessly Synchronized TDOA. Nav. Pos. T..

[13] 802.1AS-2020 (2020). IEEE Standard for Local and Metropolitan Area Networks—Timing and Synchronization for Time-Sensitive Applications.

[14] Yin Y. (2019). Research on high precision wireless time synchronizations technology for TC-OFDM. J. Beijing Univ. Posts Telecommun..

[15] Hao W., Zhongliang D., Jun M., Buyun J. Wireless synchronization technology based on ultra-wideband wireless communication technology. Proceedings of the 10th China Satellite Navigation Annual Conference.

[16] Zhao T., Zhao K., Yu C., Dong D., Zheng Z., Zhang Y. Application of Differential Time Synchronization in Indoor Positioning. Proceedings of the 2019 11th International Conference on Wireless Communications and Signal Processing (WCSP).

[17] Amar A., Leus G. A reference-free time difference of arrival source localization using a passive sensor array. Proceedings of the 2010 IEEE Sensor Array and Multichannel Signal Processing Workshop.

[18] Schau H.C., Robinson A.Z. (1987). Passive source location employing intersecting spherical surfaces from time-of-arrival difference. IEEE Trans. Acoust. Speech Signal Process..

[19] Smith J., Abel J. (1987). The spherical interpolation method of source localization. IEEE J. Ocean. Eng..

[20] Chan Y., Ho K. (1994). A simple and efficient estimator for hyperbolic location. IEEE Trans. Signal Process..

[21] Noroozi A., Sebt M.A. (2016). Weighted least squares target location estimation in multi-transmitter multi-receiver passive radar using bistatic range measurements. IET Radar Sonar Navig..

[22] Li X., Wang Q., Liu Y., Tsiftsis T.A., Ding Z., Nallanathan A. (2020). UAV-Aided Multi-Way NOMA Networks With Residual Hardware Impairments. IEEE Wirel. Commun. Lett..

[23] Yan J., Tiberius C., Bellusci G., Janssen G. Feasibility of Gauss-Newton method for indoor positioning. Proceedings of the 2008 IEEE/ION Position, Location and Navigation Symposium.

[24] Dong D., Wang M., Chen W., Zeng Z., Song L., Zhang Q., Cai M., Cheng Y., Lv J. (2016). Mitigation of multipath effect in GNSS short baseline positioning by the multipath hemispherical map. J. Geod..

[25] Wei X., Wang L., Wan J. A New Localization Technique Based on Network TDOA Information. Proceedings of the 2006 6th International Conference on ITS Telecommunications.

[26] Pierrot J.-B. Time Synchronization in UWB Ad Hoc Networks Using TOA estimation. Proceedings of the 2005 IEEE International Conference on Ultra-Wideband.

[27] Dezhang C., Hao T., Jida W. (2011). Research of TDOA cooperative location algorithm based on Chan and Tylor. Comput. Sci..

[28] Giancarlo F., Andreas M., Linda S., Zekavat S.A., Michal B.R. (2019). Anchor Placement. Handbook of Position Location—Theory, Practice and Advances.

[29] Jinyu X., Wei W., Zhongliang Z. (2004). A new TDOA algorithm based on Taylor series expansion in cellular networks. J. Com..

